# Neuregulin-1 correlates to early castration-resistant prostate cancer after prostate cancer patients receiving androgen deprivation therapy

**DOI:** 10.7150/jca.112954

**Published:** 2025-07-11

**Authors:** Siyu Li, Wenyang Zhao, Dan Wang, Lei Chen, Yigeng Feng, Peng Sun

**Affiliations:** Surgical Department I (Urology Department), LONGHUA Hospital Shanghai University of Traditional Chinese Medicine, No. 725 Wanping Road South, Xuhui District, Shanghai 200032, China.

**Keywords:** androgen deprivation therapy, castration-resistant, prostate cancer, PSA

## Abstract

**Background:** Neuregulin-1 (NRG1) levels were elevated in prostate cancer patients receiving androgen deprivation therapy (ADT). However, it remains unclear whether NRG1 levels could predict castration-resistant prostate cancer (CRPC) progression.

**Methods:** Prostate cancer patients were divided into CRPC and NCRPC (non-CRPC) groups. Baseline clinicopathological characteristics and prostate-specific antigen (PSA) levels were compared among two groups. Subsequently, the levels of NRG1 in blood and tumor tissue were detected using enzyme-linked immunosorbent assay, western blotting, and qPCR. The predictive value of NRG1 was evaluated using receiver operating characteristic (ROC) analysis. Meanwhile, the correlation of NRG1 with Gleason score and PSA levels was analyzed using Spearman analysis.

**Results:** The comparison analysis showed that TNM classification, Gleason scores, and PSA levels were significantly correlated to CRPC. Moreover, the serum NRG1 and the protein and mRNA levels of NRG1 were higher in CRPC patients than in NCRPC patients. ROC analysis unveiled that NRG1 levels in the patients before ADT could predict CRPC progression. Moreover, Spearman analysis also showed that NRG1 was correlated to Gleason scores and PSA levels.

**Conclusions:** Serum NRG1, NRG1 protein, and NRG1 mRNA in tumor tissue from prostate cancer patients before ADT could predict the incidence of CRPC in patients receiving ADT.

## Introduction

Prostate cancer (PC) is the most prevalent cancer and the second leading cause of cancer mortality among men worldwide 1, 2. Research indicates that with the improvement of diet and lifestyle patterns in China, particularly in economically developed areas, the number of prostate cancer patients is increasing 3. It may potentially become the leading cause of male cancer in China, posing a serious threat to male health. Since the critical role of androgens in stimulating prostate cancer growth was established, androgen deprivation therapy (ADT) has become the most common concomitant therapy for prostate cancer 4, 5. However, androgen deprivation resistance is a significant cause of prostate cancer metastasis and mortality post-treatment 6-8. Once patients experience disease progression despite hormonal manipulation, the median survival of patients with castration-resistant prostate cancer (CRPC) is 15-36 months depending on tumor characteristics 9. Therefore, it is crucial to accurately predict the progression of patients to CRPC at an early stage to develop individualized treatment plans.

The Gleason score is the internationally recognized standard for prostate cancer grading, with higher scores indicative of poorer tumor differentiation 10, 11. In localized prostate cancer, it is a significant independent prognostic factor 12. In metastatic hormone-sensitive prostate cancer (mHSPC), a Gleason score (GS) ≥ 8 is associated with an adverse prognosis 13. However, in CRPC, the prognostic utility of the Gleason score is reduced 12, 14, underscoring the need for integrating molecular biomarkers and advanced imaging modalities to enhance prognostic precision. Prostate-specific antigen (PSA) is the primary serum biomarker for prostate cancer, utilized for early detection, diagnosis, and monitoring post-treatment 15, 16. Baseline PSA levels are predictive of the lifetime risk of prostate cancer, including clinically significant and metastatic disease 17. In patients with non-metastatic castration-sensitive prostate cancer (nmCSPC), PSA dynamics following ADT initiation, including PSA rebound or sustained elevation, are prognostic for the risk of progression to CRPC and prostate cancer-specific mortality 18, 19. However, in some CRPC patients, radiographic progression may occur without a corresponding rise in PSA, requiring the use of alternative biomarkers or imaging modalities for comprehensive assessment, as PSA may not reliably reflect disease status in 20%-30% of cases, particularly in patients with low PSA secretion 20-22.

Recent studies have indicated that prostate cancer patients who receive ADT experience an increase in the secretion of neuregulin-1 (NRG1) by tumor-associated fibroblasts within tumor tissues 23. Neuregulins (NRGs) are a subclass of the epidermal growth factor family because they were initially identified as activators of the ERBB2 receptor tyrosine kinase 2 24. NRG1 principally acts as a ligand by binding to ERBB3/HER3 and ERBB4/HER4, leading to the stimulation of diverse pathways, including mitogen-activated protein kinase, protein kinase C, signal transducer and activator of transcription, and phosphatidylinositol-4,5-bisphosphate 3-kinase/Akt serine/threonine kinase signaling pathways, which regulates cell proliferation and differentiation in multiple organs 25. Under androgen receptor (AR) suppression, NRG1 activates AKT via ErbB3 phosphorylation, inhibiting AR degradation and promoting its nuclear translocation, thereby enhancing the transcription of AR target genes 26, 27. In certain CRPC patients, NRG1 overexpression is linked to the generation of AR splice variants, which lack the ligand-binding domain but retain transcriptional activity, leading to resistance to androgen deprivation therapy 28. Furthermore, NRG1 also activates tumor-associated fibroblasts (CAFs) or immune cells through paracrine mechanisms, promoting local androgen synthesis or inflammatory cytokine release, thus indirectly sustaining AR activity 29. During epithelial-mesenchymal transition (EMT), NRG1 regulates transcription factors like ZEB1, enhancing tumor cell migration and invasion, which are key drivers of CRPC metastasis and resistance 30. *In vitro*, NRG1 knockdown inhibits CRPC cell proliferation and increases sensitivity to enzalutamide 31. In animal models, NRG1 inhibitors combined with AR antagonists effectively delay CRPC progression 32. Clinically, elevated NRG1 expression correlates with poor prognosis and shorter progression-free survival in CRPC patients, particularly in those with low AR expression subtypes 33. NRG1 promotes androgen resistance and CRPC progression through multiple mechanisms, including bypassing AR signaling and modulating the tumor microenvironment. These findings suggest that NRG1 could serve as a potential predictive biomarker for CRPC progression in prostate cancer patients undergoing therapy. Therefore, our study aims to analyze the value of serum NRG1 levels in prostate cancer patients before ADT, as well as NRG1 levels in biopsy tissues and NRG1 gene expression, for predicting CRPC progression within one year of ADT.

## Materials and Methods

### Enrolled patients

This study enrolled a total of 259 patients meeting the criteria, of which 18 were lost to follow-up and 4 died from other causes within a year. After exclusions, 85 of the remaining 237 patients developed CRPC within a year, while 152 did not. Of the 85 CRPC patients, 31 died of prostate cancer within a year, compared to 16 of the 152 patients who did not develop CRPC. The study was approved by LONGHUA Hospital, and written informed consent was derived from the participants.

### Diagnostic criteria for CRPC

The diagnostic criteria for CRPC adopt the guideline published by the European Association of Urology in 2016, which defines serum testosterone levels less than 50 ng/mL (1.7 nmol/L); biochemical recurrence (i.e., continuous ≥ 3 times, with a gap < 1 week; PSA elevation by ≥ 50% from the lowest levels, with ≥ 2 times); and the emergence of new metastases or an expansion of infiltration areas.

### Inclusion criteria

Patients would be eligible for inclusion in our study if they met the following criteria: (1) a tissue diagnosis of prostate cancer via prostate biopsy or urethral electro-prostatectomy, (2) T2-T4 stage with locally advanced or metastatic disease, (3) initial treatment with endocrine therapy, (4) regular follow-up for at least one year after starting endocrine therapy, and (5) informed consent.

### Exclusion criteria

Patients with the following characteristics will be excluded from the study: (1) Patients with other tumors, immune system diseases, and important organ dysfunction such as heart, liver, and kidney; (2) Patients who have undergone radical treatment before receiving endocrine therapy, such as prostate cancer radical surgery or radical radiotherapy, as it may affect the outcome of endocrine therapy; (3) Patients who do not cooperate with treatment; (4) Patient has congenital defects such as cryptorchidism and unilateral testicle; (5) Patients with acute or chronic infectious diseases, systemic inflammatory disease.

### Treatment approach for PC patients

All patients received a unified treatment approach, consisting of medication for castration combined with anti-androgen therapy. If endocrine therapy failed, second-line endocrine therapy was performed. If second-line endocrine therapy failed, chemotherapy or symptomatic treatment was performed. The medication castration plan was as follows: goserelin injection (x19990231, Astra Zeneca, UK) was administered subcutaneously at a dose of 3.6 mg/time, once every 28 days. The anti-androgen regimen was as follows: bicalutamide tablet (J20150050, Astra Zeneca, UK) was administered orally at a dose of 50 mg/time, once per day. The patient was followed up for one year after initial treatment. The first follow-up occurred one month after initial treatment, with subsequent follow-ups every three months thereafter. Unless there were special circumstances, follow-ups were conducted either in outpatient visits or by phone. The endpoint event was defined as either the patient developing CRPC or death.

### Measurement of serum PSA

Digital rectal examination, catheterization, transrectal ultrasound, or prostate massage should be avoided before sampling. 5 mL of venous blood is collected from fasting patients, and the serum is immediately separated by centrifugation at 3000 rpm for 10 minutes. The serum was stored at -80 ℃ before detection. The levels of PSA are determined using the fully automatic chemiluminescence instrument (cobas e601, Roche, Penzberg, Germany).

### Enzyme-linked immunosorbent assay (ELISA)

The tumor tissue was subjected to lysis using Radio Immunoprecipitation Assay buffer (R0278, Sigma, MO, Germany), and the protein was collected by centrifugation at 13,000 rpm for 10 minutes. The NRG1 levels from serum and biopsy tissue were determined using an ELISA kit (EH1972, Fine Biotechnology, Wuhan, China). The detailed process was according to the manufacturer's described protocols.

### RT-qPCR

Total RNAs were isolated from the puncture biopsy tissue using Trizol reagent (Thermo, Shanghai, China) following the manufacturer's instructions. cDNA was synthesized using a commercial cDNA kit (Vazyme, Nanjing, China). RT-PCR was performed using the SYBR green PCR master mix (Yeasen, Shanghai, China) with the following primers. *NRG1:* the forward primer CGGTGTCCATGCCTTCCAT (5' to 3') and reverse primer GTGTCACGAGAAGTAGAGGTCT; *GAPDH*: the forward primer TGTGGGCATCAATGGATTTGG and reverse primer ACACCATGTATTCCGGGTCAAT. The *GAPDH* was the internal control for normalization.

### Statistical analysis

Statistical analysis was performed using SPSS software version 26.0. Data are presented as mean ± standard deviation (SD), and p-values are reported in tables and corresponding figures. p < 0.05 indicates statistical significance. Comparisons between the two groups used Fisher's exact test or Chi-square test. For the receiver operating characteristic (ROC) analysis, the cutoff value corresponding to the maximum Youden index was selected, as it provided the best balance between missed diagnoses (false negatives) and misdiagnoses (false positives). P-values less than 0.05 were considered statistically significant.

## Results

### Baseline clinicopathological characteristics of prostate cancer patients

Among 237 enrolled patients, 85 cases developed into CRPC within one year, while 152 cases did not. The baseline characters including age, TNM classification, Gleason score, ECOG score, and Osseous metastasis were collected and PSA levels were detected. Consequently, an analysis was conducted to investigate the incidence of CRPC in prostate cancer patients undergoing ADT within one year. The retrospective analysis revealed significant differences in TNM staging, Gleason score, and pre-treatment serum PSA levels between the NCRPC and CRPC groups (Table 1).

### NRG1 was elevated in CRPC patients

**Figure 1:**
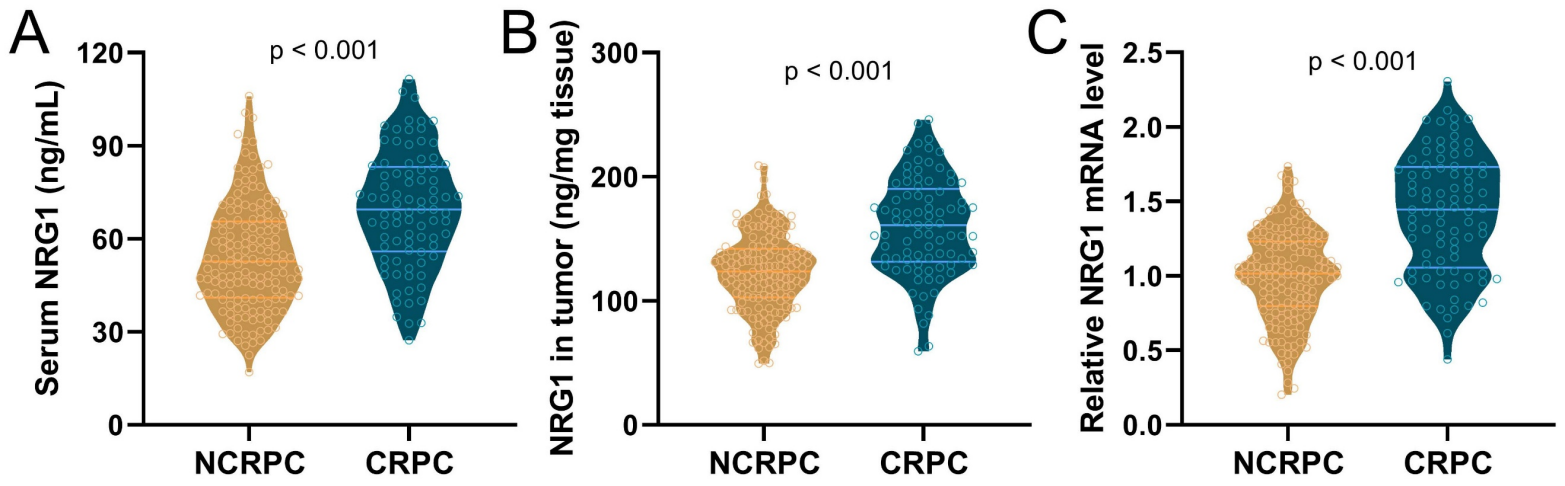
Comparisons of serum NRG1 levels (**A**), NRG1 levels in tumor (**B**) and relative NRG1 mRNA expressions in tumor (**C**) between prostate cancer patients got CRPC (n = 85) or NCRPC (n = 152) within 1 year after ADT. p values were calculated from the unpaired t-test with Welch's correction.

Among the 85 patients who progressed to CRPC, and 152 patients who did not progress to CRPC, the levels of NRG1 in serum and biopsy tissues, as well as the relative NRG1 mRNA levels in tumor tissue, were detected before the initiation of ADT. The results showed that the levels of serum NRG1 (Fig. 1A), NRG1 protein in tumor tissues (Fig. 1B) and NRG1 mRNA in tumor tissue (Fig. 1C), were all significantly higher in CRPC patients before receiving ADT treatment, compared to NCRPC patients.

### ROC analysis for the predictive values of NRG1

**Figure 2:**
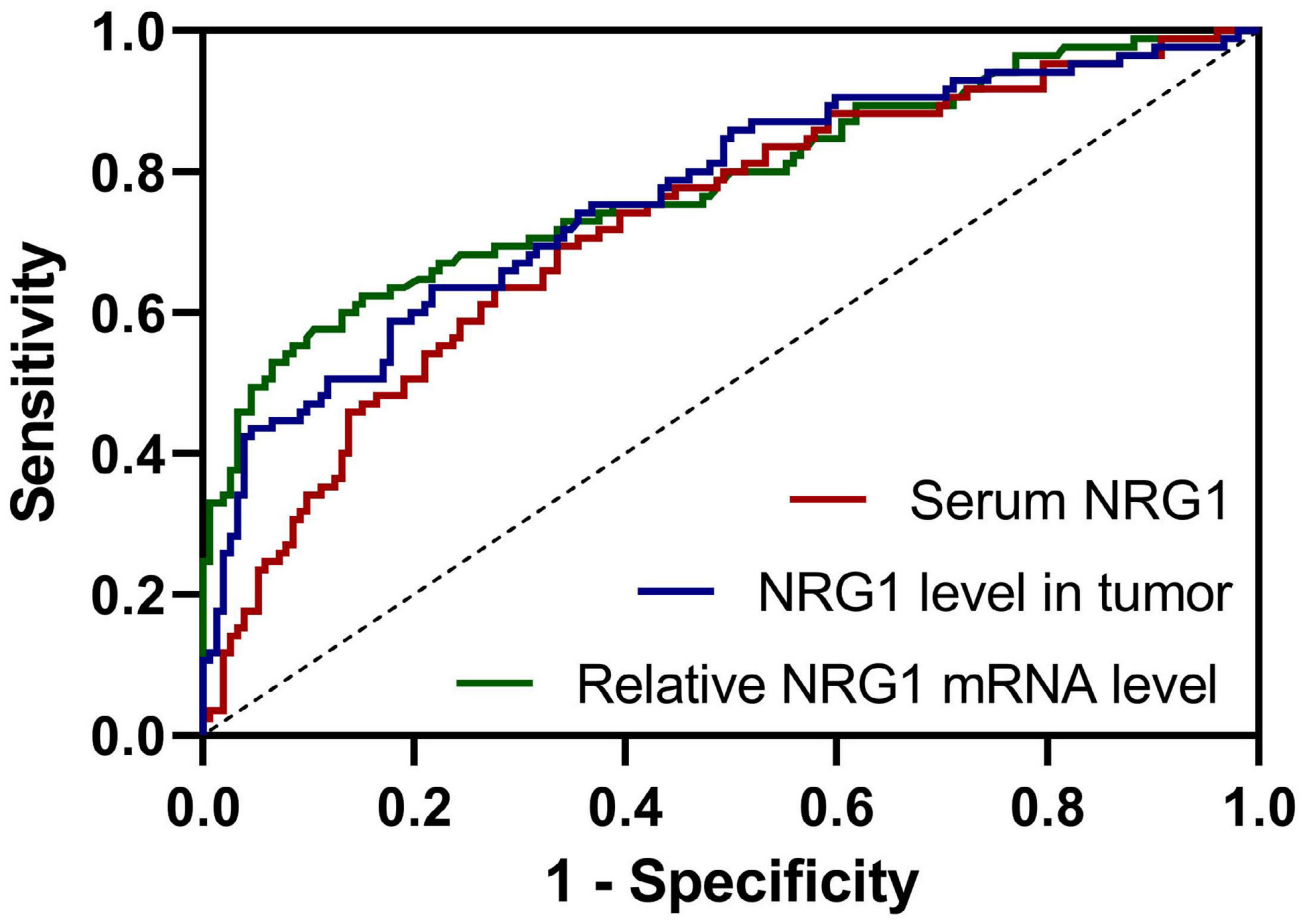
ROC analysis for the predictive values of serum NRG1 levels, NRG1 levels in tumor and relative NRG1 mRNA expressions in tumor for prostate cancer patients got CRPC within 1 year after ADT.

The value of NRG1 levels in serum, biopsy tissue, and relative NRG1 mRNA in tumor tissue of PC patients before ADT for predicting CRPC progression within one year after ADT was investigated using ROC analysis. The ROC curve analysis revealed that relative NRG1 mRNA expression in tumor tissue exhibited the highest diagnostic accuracy for predicting CRPC progression, with an AUC of 0.78 (95% CI: 0.72-0.85), sensitivity of 57.65%, and specificity of 89.47% (Fig. 2 and Table 2). The cut-off value for relative NRG1 mRNA expression was set at 1.353, and the corresponding Youden index was 0.47, indicating the optimal balance between sensitivity and specificity. Meanwhile, NRG1 levels in tumor tissue also demonstrated robust predictive performance, with an AUC of 0.77 (95% CI: 0.70-0.83), sensitivity of 62.35%, and specificity of 78.29%, with a cut-off value of 143.8 ng/mL and a Youden index of 0.42 (Fig. 2 and Table 2). Similarly, serum NRG1 levels displayed moderate discriminatory power, with an AUC of 0.72 (95% CI: 0.66-0.79), sensitivity of 63.53%, specificity of 72.37%, and a cut-off value of 64.1 ng/mL, yielding a Youden index of 0.36 (Fig. 2 and Table 2). These findings underscore that while relative NRG1 mRNA expression in tumor tissue provides the most accurate prediction, both tumor NRG1 protein levels and serum NRG1 levels offer complementary diagnostic value in the prediction of CRPC risk of prostate cancer after ADT.

### The correlation of NRG1 levels in different tissues

**Figure 3:**
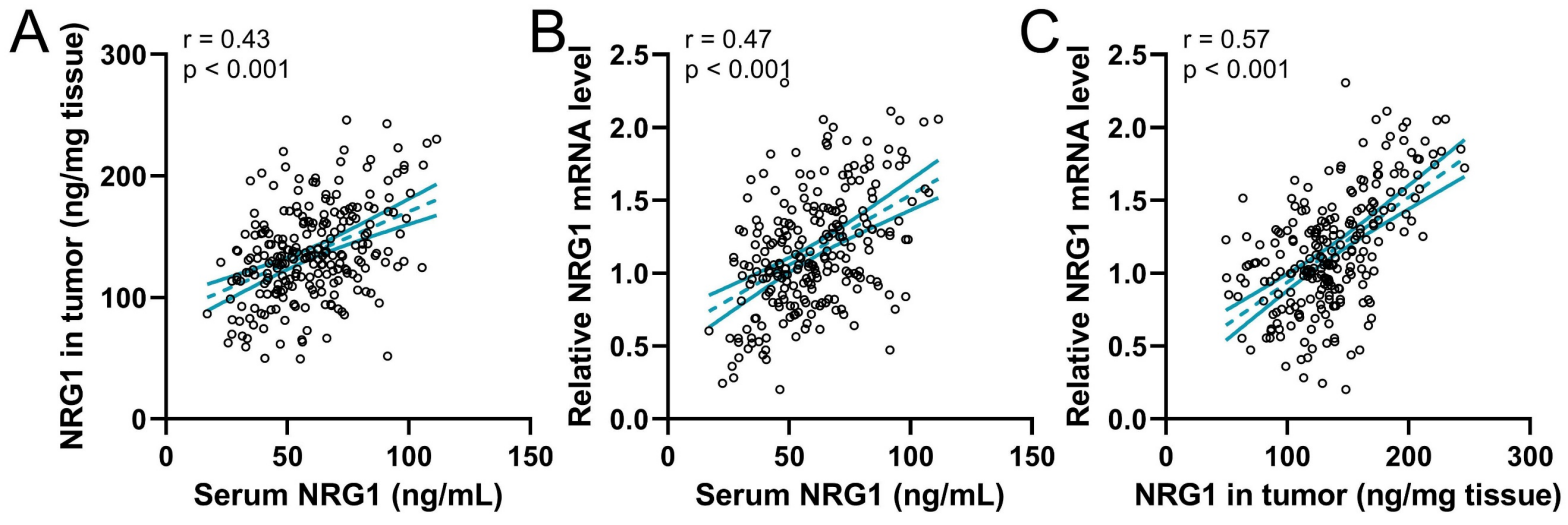
Pearson correlation analysis of serum NRG1 levels with NRG1 levels in tumor (**A**), serum NRG1 levels with relative NRG1 mRNA expressions in tumor (**B**) and NRG1 levels in tumor with relative NRG1 mRNA expressions in tumor (**C**) in prostate cancer patients (n = 237).

The correlation among serum NRG1, NRG1 in biopsy tumor, and relative NRG1 mRNA levels in tumor tissue were analyzed using Pearson analysis. The result revealed that the serum NRG1 levels were positively correlated to NRG1 in biopsy tumor (Fig. 3A). Meanwhile, the serum NRG1 levels were also positively correlated to the relative NRG1 mRNA levels in tumor tissue (Fig. 3B). Moreover, NRG1 in biopsy tumor was strongly correlated to the relative NRG1 mRNA levels in tumor (Fig. 3C). These findings proved that these three factors were positively correlated.

### The correlation of NRG1 with the Gleason score

**Figure 4:**
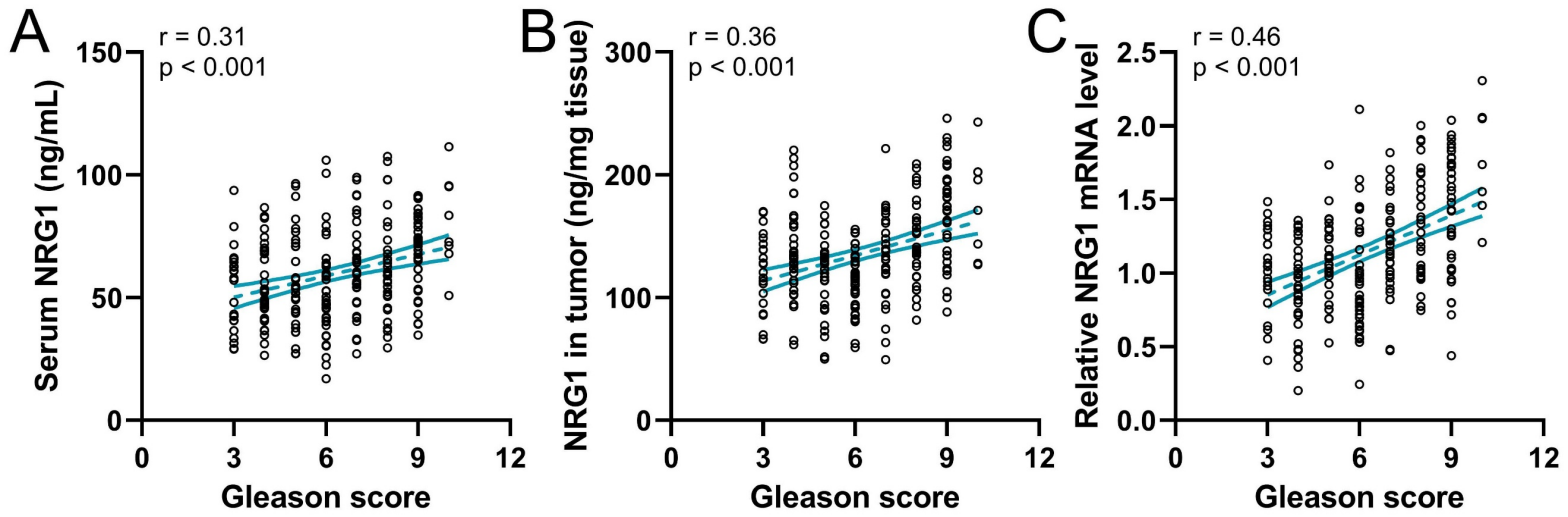
Spearman correlation analysis of Gleason scores with serum NRG1 levels (**A**), NRG1 levels in tumor (**B**) and relative NRG1 mRNA expressions in tumor (**C**) in prostate cancer patients (n = 237).

Gleason score plays a critical role in predicting patient outcomes; attempts have been made to refine histologic classification and reporting in prostate cancer to facilitate patient risk stratification 34. Therefore, the correlation of the Gleason score to NRG1 in 237 prostate cancer patients before ADT was analyzed. The result revealed that the serum NRG1(Fig. 4A), the NRG1 protein and mRNA levels in tumor tissue (Fig. 4B-C) were positively correlated to the Gleason score, indicating the NRG1 levels were associated with the outcome of PC patients.

### The correlation of NRG1 with serum PSA levels

**Figure 5:**
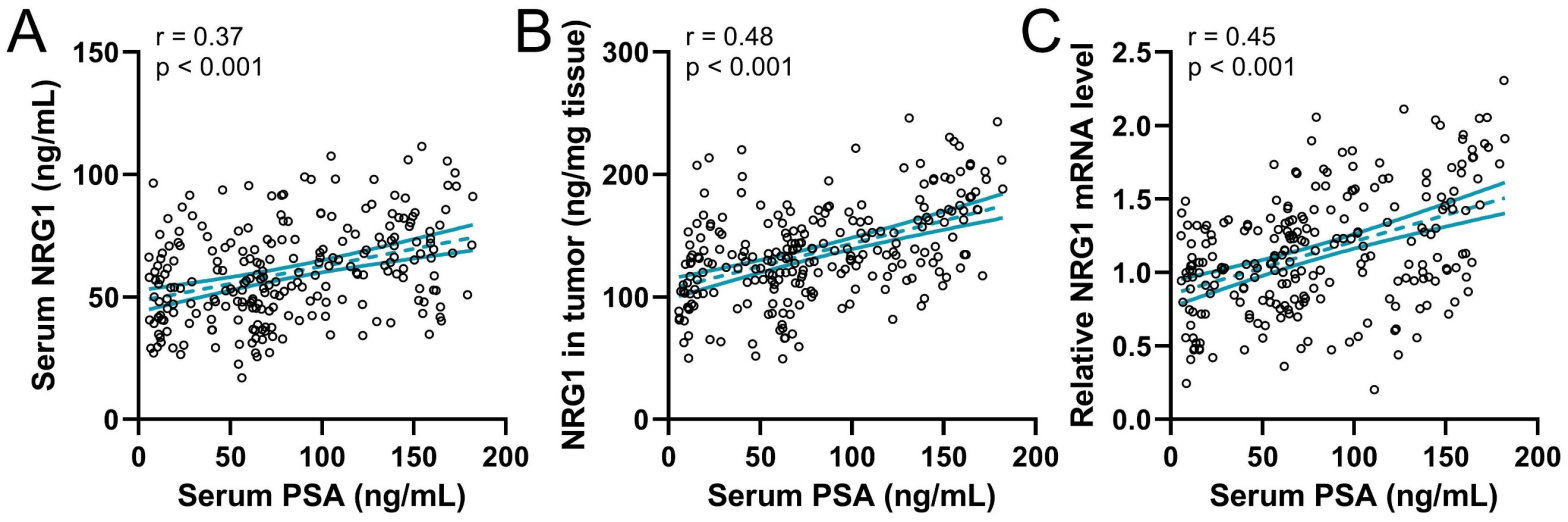
Spearman correlation analysis of serum PSA levels with serum NRG1 levels (**A**), NRG1 levels in tumor (**B**) and relative NRG1 mRNA expressions in tumor (**C**) in prostate cancer patients (n = 237).

Previous data showed that serum PSA levels were associated with CRPC, therefore the correlation between serum PSA levels and NRG1 levels in prostate cancer before ADT was explored. The analysis result showed that the serum PSA levels were positively associated with the serum NRG1 (Fig. 5A), NRG1 in biopsy tumor tissue (Fig. 5B) and relative NRG1 mRNA levels in tumor tissue (Fig. 5C). The PSA levels are also associated with the prognosis and progression of CRPC in PC patients, therefore, our results further suggest the correlation between NRG1 levels and the progression of CRPC in prostate cancer patients.

## Discussion

This study is the first to evaluate the predictive value of NRG1 in prostate cancer patients before ADT, for predicting CRPC progression during ADT within one year. We found that serum NRG1, NRG1 protein, and mRNA were significantly elevated in PC patients with CRPC compared to PC patients with non-CRPC. Moreover, NRG1 levels could predict CRPC incidence in patients receiving ADT. Importantly, NRG1 was also positively correlated to Gleason score and serum PSA levels, which are predictors of outcome in PC patients.

ADT is the standard treatment for patients with locally advanced or metastatic disease and biochemical recurrence 35, providing initial benefit but most develop castration-resistant prostate cancer within 2-3 years 36. The treatment of metastatic CRPC is currently limited by the inadequate therapeutic effect of the current treatment options, which target a single mechanism. This presents a significant challenge for clinicians in managing metastatic CRPC patients 37, 38. It is crucial to investigate high-risk factors for the progression of advanced prostate cancer into CRPC. Ma et al found that the M stage of PC was positively correlated to CRPC 39, however, our study revealed that the TNM stages were not correlated to CRPC progression.

Castration resistance is defined as either clinical progression, such as the development of metastatic disease or progression of pre-existing disease, or biochemical progression, characterized by three consecutive rises in PSA levels above nadir, in the presence of castrate levels of circulating testosterone 40. Numerous reports have demonstrated the effectiveness of utilizing a decline in PSA levels following the initiation of ADT to predict the risk of progression to CRP 41, 42. Regular monitoring of PSA aids the identification of high-risk patients and improves treatment outcomes. According to the study conducted by Keto et al., men with a PSA nadir (the lowest levels of PSA a patient reaches after treatment) of 0.01-0.2 ng/mL have a five-fold increased risk of developing CRPC compared to men with undetectable PSA nadir. Our study detected the PSA levels in PC patients before ADT, it was found that the percentage of patients with high PSA levels in the CRPC group was higher than those in the NCRPC group. However, our analysis only detected PSA levels in CRPC but did not evaluate the variation of PSA levels in PC patients before and after ADT. These findings demonstrated the variation of PSA levels could predict the CRPC progression of the PC patients receiving ADT, however, there are some limitations to using PSA nadir as a predictor since the time point at which it occurs can vary. Therefore, it was critical to screen a suitable predictor for CRPC progression.

The NRG1 gene is located on chromosome 8 in region 8p12, encoding a growth factor called NRG1 that contains an epidermal growth factor-like domain. This domain binds to human tyrosine kinases of the ErbB/HER receptor group, specifically ERBB3 and ERBB4, leading to the activation of ErbB-mediated downstream signaling pathways that promote cell growth 43. The activity of soluble NRG1 (sNRG1) is a clinical clue for combating its effects because an environment rich in NRG1 drives resistance to androgen deprivation. Due to that androgen deprivation with bicalutamide or enzalutamide -each a commonly prescribed anti-androgen for patients with prostate cancer- drives additional NRG1 production 23. Most importantly, Zhang et al. showed that a combination of HER2 and HER3 blockade severely restricts tumor growth and improves response to ADT *in vivo*. Given clinical data implicating NRG1 upregulation in poor outcomes on ADT, it is tempting to speculate that patients may benefit from pre-therapy selection by NRG1 immunohistochemistry on tumor specimens or by sNRG1 detection in the blood. In our study, the serum NRG1, the NRG1 protein and mRNA in tumor tissue were detected. The analysis result showed that the levels of NRG1 from blood and tumor tissue were significantly higher in CRPC patients than that in NCRPC patients, demonstrating that NRG1 might correlate to CRPC progression.

The ROC analysis revealed that NRG1 could predict the CRPC progression. The cutoff values for NRG1 biomarkers-serum NRG1 levels (64.1 ng/mL), NRG1 levels in tumor tissue (143.8 ng/mL), and relative NRG1 mRNA expression in tumor tissue (1.353)-hold important clinical implications for predicting CRPC progression. Serum NRG1 at 64.1 ng/mL demonstrates moderate sensitivity (63.53%) and specificity (72.37%), serving as a valuable adjunctive marker, particularly in cases where PSA levels are unreliable. However, due to its moderate Youden index, it should be used in conjunction with other diagnostic tools. NRG1 levels in tumor tissue, with a cutoff of 143.8 ng/mL, exhibit higher specificity (78.29%) and sensitivity (62.35%), making it particularly useful for identifying tumors at risk of progressing to CRPC and guiding timely intervention. The cutoff for relative NRG1 mRNA expression in tumor tissue (1.353) provides the highest diagnostic performance, with specificity of 89.47% and sensitivity of 57.65%, rendering it valuable for confirming CRPC in challenging cases and refining risk stratification. Collectively, these biomarkers enhance early detection, improve prognostic accuracy, and offer potential for more personalized treatment strategies, thereby advancing clinical management of prostate cancer.

The Gleason grading system plays a crucial role in determining the prognosis of prostate cancer and in making clinical decisions. The Gleason score is entirely based on the classification of adenocarcinoma growth patterns. These patterns are assigned a Gleason grade from 1 to 5. As prostate cancer is a diverse disease, the Gleason score is determined by adding together the most common grade and the highest grade in biopsies, and the two most predominant grades in radical prostatectomy specimens 44, 45. Our analysis revealed that CRPC patients with a Gleason score above 7 were more numerous than those with a Gleason score less than 7, indicating Gleason score was positively correlated with CRPC progression. More importantly, spearman correlation analysis of Gleason scores with NRG1 was conducted. It was found the positive correlation of Gleason scores with NRG1 was independent on the source of NRG1.The origin of sNRG1 is still a matter of debate. Although Zhang et al. have identified that CAFs are a possible source of paracrine sNRG1, data from The Cancer Genome Atlas (TCGA) dataset 46 suggest that other potential sources of NRG1 could include prostate cancer cells, benign genitourinary tract cells, immune cells, and neuroendocrine cells, whether it is autocrine, paracrine, or endocrine remained unclear. Our result showed that the soluble NRG1 from blood, and the NRG1 from tumor tissue were correlated to CRPC and could be used to predict the CRPC incidence. However, the exploration of NRG1 source is better for understanding the mechanism of NRG1 regulating CRPC.

Our study indicates that NRG1 levels can augment the predictive value of PSA and Gleason score in forecasting CRPC progression in prostate cancer patients. While PSA and Gleason score are critical prognostic markers, they exhibit certain limitations in specific patient populations. Elevated NRG1 levels in CRPC patients showed a positive correlation with both PSA and Gleason score, suggesting that NRG1 may enhance the prognostic accuracy of these conventional biomarkers. NRG1 may serve as a more reliable indicator of disease progression, particularly in cases where PSA levels are low or ambiguous. Moreover, its association with the Gleason score highlights its potential to refine risk stratification, particularly in patients with intermediate Gleason scores. Integrating PSA, Gleason score, and NRG1 levels might provide a more comprehensive and precise risk assessment for CRPC, facilitating more tailored treatment strategies.

However, this study still has several limitations that should be addressed. Firstly, the relatively small sample size may limit the generalizability of the findings, and larger studies are needed to confirm the results and the robustness of NRG1 as a predictive biomarker for CRPC. Moreover, patient sample heterogeneity, including variations in clinical characteristics and treatment responses, could introduce confounding factors. Additionally, the single-center nature of the study also limits the applicability of the findings to broader populations, highlighting the need for multi-center validation. Finally, challenges in clinical implementation, such as standardizing NRG1 measurement techniques and integrating it into routine practice, need to be addressed.

## Conclusion

Our study found that NRG1 levels were higher in CRPC patients compared to NCRPC patients, regardless of whether NRG1 was sourced from blood or tumor tissues. Importantly, NRG1 levels were positively correlated to PSA levels and Gleason grading, indicating that NRG1 levels from the PC patients before ADT could predict CRPC progression. The ROC analysis makes it convincing that the serum NRG1, the protein and the mRNA levels of NRG1 in tumor tissue could predict the CRPC progression with high sensitivity and specificity.

## Figures and Tables

**Table 1:** Baseline clinicopathological characteristics of prostate cancer patients with castration resistant prostate cancer (CRPC) and without (NCRPC) within 1 year after androgen deprivation therapy

Factors	NCRPC (n = 152)	CRPC (n = 85)	p value
Age (years)			
< 70	78 (51.3 %)	37 (43.5 %)	0.279
≥ 70	74 (48.7 %)	48 (56.5 %)	
T classification			
T2	51 (33.5 %)	21 (24.7 %)	0.001
T3	72 (47.4 %)	29 (34.1 %)	
T4	29 (19.1 %)	35 (41.2 %)	
N classification			
N0	94 (61.8 %)	38 (44.7 %)	0.014
N1	58 (38.2 %)	47 (55.3 %)	
M classification			
M0	70 (46.1 %)	26 (30.6 %)	0.027
M1	82 (53.9 %)	59 (69.4 %)	
Gleason score			
< 6	71 (46.7 %)	18 (21.2 %)	< 0.001
6-7	45 (29.6 %)	27 (31.8 %)	
> 7	36 (23.7 %)	40 (47.0 %)	
ECOG score			
≤ 2	26 (17.1 %)	23 (27.1 %)	0.094
> 2	126 (82.9 %)	62 (72.9 %)	
Osseous metastasis			
Yes	54 (35.5 %)	34 (40 %)	0.192
No	98 (64.5 %)	51 (60 %)	
PSA (ng/mL)			
< 20	29 (19.1 %)	10 (11.8 %)	< 0.001
20-100	86 (56.6 %)	32 (37.6 %)	
> 100	37 (24.3 %)	43 (50.6 %)	

The data were shown as n (percentage). The comparisons of data between the two groups were done Fisher's exact test or Chi-square test. TNM: Tumor Node Metastasis, ECOG: Eastern C

**Table 2:** Results of ROC analysis for the predictive values of serum NRG1 levels, NRG1 levels in tumor and relative NRG1 mRNA expressions in tumor for prostate cancer patients with castration resistant prostate cancer (CRPC) within 1 year after androgen deprivation therapy

	Cut-off	AUC (95% CI)	p	Sensitivity (%)	Specificity% (%)	Youden index
Serum NRG1 levels	64.1 ng/mL	0.72 (0.66 to 0.79)	< 0.001	63.53	72.37	0.36
NRG1 levels in tumor	143.8 ng/mL	0.77 (0.70 to 0.83)	< 0.001	62.35	78.29	0.42
Relative NRG1 mRNA expressions in tumor	1.353	0.78 (0.72 to 0.85)	< 0.001	57.65	89.47	0.47

CI: confidence interval.
